# Exploring Antibiotic Resistance Patterns in *Escherichia coli* Isolates from Urinary Tract Infections: A Retrospective Study

**DOI:** 10.3390/jcm14093196

**Published:** 2025-05-05

**Authors:** Rama Alkhawaldeh, Lobna Gharaibeh, Amer Hayat Khan, Khawla Abu Hammour, Mohammed Zawiah, Sireen AR. Shilbayeh, Rana K. Abu-Farha

**Affiliations:** 1 Discipline of Clinical Pharmacy, School of Pharmaceutical Sciences, Universiti Sains Malaysia, Pulau 11700, Penang, Malaysia; ramabaker73@gmail.com (R.A.);; 2 Biopharmaceutics and Clinical Pharmacy Department, Faculty of Pharmacy, Al-Ahliyya Amman University, Amman 19111, Jordan; 3 Department Biopharmaceutics and Clinical Pharmacy, Faculty of Pharmacy, The University of Jordan, Amman 11942, Jordan; 4 Department of Clinical Practice, College of Pharmacy, Northern Border University, Rafha 91431, Saudi Arabia; 5 Department of Pharmacy Practice, College of Pharmacy, Princess Nourah bint Abdulrahman University, Riyadh 11671, Saudi Arabia; 6 Clinical Pharmacy and Therapeutics Department, Faculty of Pharmacy, Applied Science Private University, Amman 11937, Jordan

**Keywords:** susceptibility test, empiric antibiotic, *Escherichia coli*, urinary tract infections, antibiotic resistance

## Abstract

**Objectives**: This retrospective analysis was conducted to update the antibiotic sensitivity profiles of *Escherichia coli (E. coli)* in urinary tract infections (UTIs) among adults (≥18 years) in Jordan. **Methods**: Data were collected from patients diagnosed with UTIs and confirmed by positive *E. coli* cultures between 1 January 2019 and 9 July 2021. The resistance patterns of *E. coli* to empiric antibiotics were assessed by matching the prescribed antibiotics with those tested, using clinical breakpoints to classify isolates as susceptible or resistant. **Results**: During the study, 230 patients with urine cultures confirming *E. coli* infection were included. Empiric antibiotics were prescribed to 87.4% (n = 201) of patients. Sensitivity testing was performed for 136 patients (59.1%), revealing that 62 patients (27%) had *E. coli* strains resistant to at least one antibiotic. Among the 236 empiric antibiotics prescribed, the appropriateness of 72 agents (30.5%) could not be determined owing to the absence of sensitivity testing. The culture results indicate that eight antibiotics (3.3%) did not provide adequate coverage against *E. coli*. Of the remaining 156 agents (66.1%) subjected to sensitivity testing, 89 (37.7%) were effective, while 67 (28.3%) were ineffective owing to bacterial resistance. Patients who underwent sensitivity tests performed had significantly longer hospital stays (10 days) than those who did not (8 days; *p* = 0.032). Female patients had a higher proportion of resistant *E. coli* isolates (51.5%) compared to males (30.8%, *p* = 0.036). **Conclusions**: This study highlights the importance of ongoing surveillance to guide empiric therapy and promote appropriate antibiotic use. Tailoring treatment based on patient-specific factors is essential to effectively managing antimicrobial resistance and improving clinical outcomes.

## 1. Introduction

Urinary tract infections (UTIs) rank as the second most prevalent bacterial infections in primary care [1]. This condition affects individuals across all age groups and sexes. However, the primary risk factors often include female anatomy, inadequate personal hygiene practices, pregnancy, urinary tract blockages, prolonged catheter use, urethral reflux, and spermicidal contraception [2]. Moreover, UTIs can lead to temporary discomfort, such as elevated temperature, dysuria, and lower abdominal aches, potentially resulting in chronic kidney failure [3].

Gram-positive bacteria, including *Enterococcus* and *Staphylococcus saprophyticus*, contribute to UTIs [4]. Additionally, Gram-negative bacteria such as *Klebsiella* and *Enterobacter* species are commonly implicated in these infections [5]. These pathogens are continuously developing resistance to frequently used traditional medications, as well as newer, more potent antimicrobial substances [6]. *Escherichia coli (E. coli)* is considered the primary causative agent of UTIs [7]. Similarly to other members of the Enterobacteriaceae family, it employs sophisticated strategies to evade lethal drug doses, including enzyme degradation, the modification of drug targets, reduced absorption, and the upregulation of efflux proteins [8].

Researchers worldwide have reported rising antimicrobial resistance in *E. coli* isolates [1]. A key factor contributing to this resistance is *E. coli*’s production of extended-spectrum beta-lactamases (ESBLs), which degrade third-generation cephalosporins and aztreonam; however, these enzymes are inhibited by clavulanic acid [9]. Furthermore, UTIs caused by multidrug-resistant (MDR) bacteria, when treated with ineffective empirical antibiotics, can result in severe complications, such as sepsis, elevated mortality rates, increased treatment costs, prolonged hospitalization, and reduced productivity [10].

Urine culture and susceptibility testing are widely recognized as the gold standards for diagnosing and managing UTIs [11]. While culture results determine infection prevalence, sensitivity testing evaluates the response of germs to different antibiotics [12]. Delays in susceptibility testing often lead to the implementation of rapid empirical antibiotic treatment to mitigate UTIs [9]. However, this approach promotes the growth of resistance, particularly ESBLs and multidrug resistance, at a global level. According to the French Society of Infectious Diseases, resistance rates should preferably be less than 10–20% when starting empirical treatment [9]. Therefore, recognizing current regional data on the antimicrobial susceptibility patterns of common uropathogens is essential for guiding effective treatment decisions.

To address this need, we conducted a retrospective analysis aimed at updating our understanding of *E. coli’s* recent antibiotic sensitivity profiles among adult patients with UTIs in Jordan. By shedding light on this issue, we sought to provide valuable insights into the optimization of antibiotic therapies for UTI management.

## 2. Materials and Methods

### 2.1. Study Design and Study Subjects

This retrospective observational study was performed at Jordan University Hospital (JUH), a leading academic teaching hospital in Jordan with a 550-bed capacity. JUH comprises approximately 11 departments, including specialized wards and units. The study involved adult patients aged ≥18 years who were hospitalized at JUH between 1 January 2019 and 9 July 2021. Patient samples were collected from the internal medicine department. Patients were eligible for inclusion if they were diagnosed with a UTI, classified under the ICD-10 code (N39.0), and their microbiological cultures were positive for *E. coli.* The ICD-10 code used does not differentiate between hospital-acquired or community-acquired infections. The exclusion criteria were negative microbiological cultures, a lack of culture requests, cases with other microbes identified, and urine specimens obtained after the initiation of empiric antibiotic therapy.

### 2.2. Data Collection

Data collection from computerized clinical records involved obtaining comprehensive patient information, including age, sex, admission/discharge dates, the length of hospital stay, the use of chronic medications, and the details of prescribed empiric antibiotics. Urine culture results and susceptibility data were retrieved from the electronic laboratory system at JUH.

### 2.3. Identification of Resistance Pattern for Empiric Antibiotics

The resistance profile of *E. coli* strains to empiric antibiotics was determined by analyzing susceptibility reports obtained from urine cultures. Initially, empiric antibiotics prescribed for the treatment of UTIs were identified. The panel of antibiotics tested in the susceptibility assay was then reviewed to ensure that it included the selected empiric antibiotics. Antibiotic susceptibility testing was conducted following Clinical and Laboratory Standards Institute (CLSI) guidelines [13]. Susceptibility results for each selected empiric antibiotic were interpreted based on established clinical breakpoints. Isolates were classified as susceptible or resistant based on these breakpoints. The resistance pattern analysis included the following antibiotics: ampicillin, amoxicillin–clavulanic acid, piperacillin–tazobactam, cefazolin, cefuroxime, cefotaxime, ceftriaxone, ertapenem, imipenem, meropenem, gentamicin, amikacin, levofloxacin, trimethoprim–sulfamethoxazole, and nitrofurantoin. For patients with resistant strains, whether a change was made to the empiric treatment regimen following resistance identification was assessed. Subsequent targeted therapy—which physicians adjust in light of sensitivity testing—was not assessed in this analysis.

### 2.4. Statistical Analysis

Data coding, input, and analysis were carried out using SPSS Statistics for Windows, Version 22.0 (IBM Corp., Armonk, NY, USA). Descriptive statistics were computed for continuous variables in terms of means and standard deviations, while categorical variables were represented as percentages. The normality of the continuous variables was assessed using the Shapiro–Wilk test, with a *p*-value ≤ 0.05 indicating a non-normal distribution of continuous variables. The effect of *E. coli* bacterial resistance and the presence or absence of susceptibility reporting on hospital length of stay were evaluated using a two-tailed Mann–Whitney U test. The association between sex and *E. coli* resistance was evaluated using the Pearson Chi-Square test. Statistical significance was set at *p* < 0.05.

## 3. Results

During the research period, a total of 230 urine culture samples, all positive for *E. coli*, were analyzed, with each sample collected from a different patient. No patient contributed more than one sample. The patients had a median age of 64 years (IQR = 25), and nearly half were elderly individuals aged above 65 years (n = 113, 49.1%). Female patients constituted the majority, accounting for 72.2% (n = 166) of the sample. Additionally, more than half of the patients (n = 122, 53%) were receiving polypharmacy (defined as the use of ≥4 medications), with a median hospital stay of 10 days (IQR = 9). Additional details on the demographic and medical characteristics of the study participants are provided in Table 1.

The study identified 276 microorganisms in the urine specimens (Table 2). The mean number of pathogens listed on the culture reports was 1.2 ± 0.4. All urine culture specimens indicated the presence of *E. coli*, while some samples exhibited the co-occurrence of *E. coli* with additional microorganisms per culture. Among these, 40 samples showed the presence of two microorganisms per culture, whereas 3 samples exhibited three microorganisms. The most frequently identified microorganisms following *E. coli* were *Staphylococcus aureus* (n = 16, 7.0%) and *Klebsiella pneumoniae* (n = 5, 2.2%). Empiric antibiotics were prescribed to 87.4% (n = 201) of patients. On average, these patients received 1.0 ± 0.5 empiric antibiotics (n = 236) (Table 2). Approximately 12.6% (n = 29) of patients with positive culture results received no treatment, while the majority (n = 170, 73.9%) received a single antibiotic medication. Only 27 patients received two antimicrobial medications and only 4 individuals (1.7%) received prescriptions for three agents.

**Figure 1:**
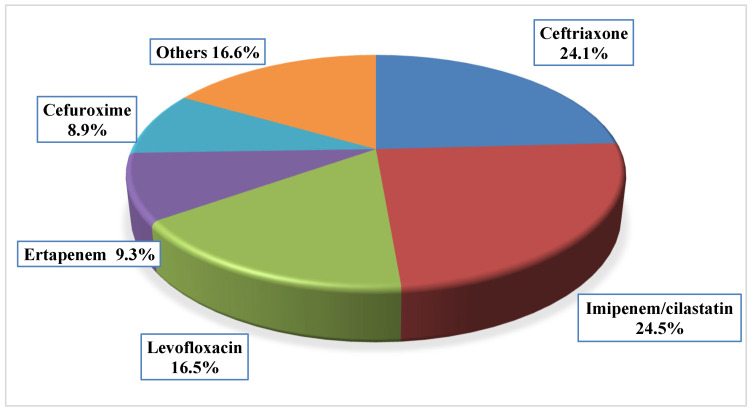
Percentage Distribution of Most Common Empiric Antibiotics Prescribed to Study Participants (n = 236 prescriptions).

Carbapenems represented the most commonly prescribed class, accounting for 33.8% of prescriptions (imipenem/cilastatin 24.5%, n = 58; ertapenem 9.3%, n = 22), followed by third-generation cephalosporins, which accounted for 24.4% (ceftriaxone 24.0%, n = 57; cefotaxime 0.4%, n = 1). Fluoroquinolones, represented by levofloxacin, comprised 16.5% (n = 39) of prescriptions. Polymyxins were rarely prescribed, with colistin accounting for 0.4% (n = 1). Additional details of the prescribed empirical antibiotics are shown in Figure 1.

In this study, among the 230 patients with *E. coli* infections, 35 patients (15.2%) either received empiric antibiotics that did not provide coverage against *E. coli* (n = 8, 3.4%) or did not receive empiric treatment at all (n = 29, 12.6%). Of the remaining 195 patients, 136 (59.1%) underwent sensitivity testing, whereas 57 (24.8%) lacked susceptibility reporting. Among the patients with sensitivity tests, 62 (27%) were identified as having *E. coli* strains resistant to at least one antibiotic. Of these 62 patients, 40 (64.5%) had their therapy adjusted based on resistance patterns, while 22 (35.5%) did not have any changes made. Further details on the susceptibility results are presented in Table 3.

As seen in Table 4, among the 236 empiric antibiotic prescriptions, the appropriateness of 72 agents (30.5%) could not be determined because of the absence of sensitivity testing. Additionally, the culture results indicated that eight antibiotics (3.4%) did not provide adequate coverage against *E. coli.* Of the remaining 156 agents (66.1%) with sensitivity testing, 89 (37.7%) were effective against *E. coli*, while 67 (28.4%) were ineffective due to bacterial resistance. The antibiotics showing resistance against *E. coli* included ceftriaxone (32 cases, 47.8%), levofloxacin (15 cases, 22.4%), cefuroxime (10 cases, 14.9%), imipenem/cilastatin (2 cases, 3.0%), and others (8 cases, 11.9%).

Analysis of the impact of antibiotic sensitivity testing on the length of hospital stay using the Mann–Whitney U test revealed significant findings (Table 5). Patients who underwent sensitivity tests had a median hospital stay of 10 days (IQR = 10.0), which was significantly longer than the 8 days (IQR = 6.0) observed in patients who did not undergo sensitivity tests (*p* = 0.032). Patients who received empiric antibiotics that did not provide coverage against *E. coli* and those who did not receive empiric treatment at all were excluded from this analysis (n = 35).

In this study, we investigated the association between sex and antibiotic resistance in patients who underwent sensitivity analysis (n = 136) (Table 6). Notably, a higher proportion of females (51.5%) were found to have resistant *E. coli* isolates compared to males (30.8%), demonstrating a statistically significant association (*p* = 0.036). However, the analysis revealed no significant disparity in the median length of hospital stay between patients infected with resistant *E. coli* isolates (11 days, IQR = 10) and those with sensitive isolates (9.5 days, IQR = 8) (*p* = 0.190).

## 4. Discussion

Antimicrobial resistance is a major challenge in global public health. This danger is on the rise, despite different strategies to slow it down. The consequences of this alarming phenomenon are reflected in higher costs and wider gaps in healthcare between developing and developed countries [14]. One of the important efforts to address this problem is collecting antimicrobial resistance surveillance information [15]. *E. coli* is an important multidrug-resistant bacterium that develops in hospitals and is mainly responsible for UTIs [16]. In Jordan, Nairoukh et al. showed that the number of multidrug-resistant *E. coli* isolates was significantly higher in hospitalized patients than in outpatients [17]. In the national antimicrobial resistance surveillance report of 2022, published by the Ministry of Health in cooperation with the WHO, *E. coli* was one of the priority antimicrobial resistance pathogens with a percentage of MDR isolates of 36% [18].

Most of the patients in our study were females with a median age of 64 years, almost half were 65 years of age, and women have a higher risk of UTI than the rest of the population owing to anatomical features such as urethral length, frequent vaginal colonization, and problems in urine flow [19]. In addition, elderly women are highly susceptible to UTIs, with a prevalence of 20% in women older than 65 years of age [20]. This suggests that with the increasing age of the population, antimicrobial resistance to UTIs will constitute a great burden on healthcare services and patient well-being.

More than half of the study had four or more comorbidities, which can be explained by the high percentage of elderly patients in the study. The risk of many chronic diseases increases with age, and the majority of the patients with these non-communicable diseases are over 65 years old [21].

Some patients did not receive empiric therapy, which is considered the first step in treatment that is modified after the results of culture sensitivity become available [22]. Inappropriate empirical antibiotic treatment was significantly associated with a higher rate of in-hospital mortality [23]. Zhu et al. revealed that inappropriate empirical antibiotic treatment was associated with a longer length of stay, lung disease, and cardiac disease [24]. However, in a prospective cohort study, Kusin et al. assessed the effect of the empiric prescription of antibiotic therapy for UTIs and concluded that the outcomes of empiric treatment of uncomplicated UTIs using nitrofurantoin were not significantly different from those of culture-based treatment [25]. Prescribing antibiotics that did not cover *E. coli* was limited (3.5%); this is lower than reported by Esparcia et al. with 29.3% of empirical antibiotic treatments being inappropriate [23] and the 48.4% reported by Zhu et al. [24]. While these differences may be due to variations in clinical settings and study populations, a key concern remains that 64.5% of patients in our study had their therapy adjusted based on sensitivity results due to resistant *E. coli* strains. However, 35.5% of patients did not have their therapy modified, despite the presence of resistant strains. A published meta-analysis found that inappropriate empirical antibiotic treatment was associated with significantly higher mortality, with a pooled adjusted odds ratio of 1.60 (95% CI, 1.37–1.86) [26]. This emphasizes the importance of optimizing therapy to minimize the risk of adverse outcomes.

Most of the *E. coli* isolates were resistant to ceftriaxone (47.8%). This percentage is comparable to the 46.1% reported in the national antimicrobial resistance surveillance report by the Ministry of Health in Jordan. Kulkarni et al. identified a 66.6% rate of resistance to ceftriaxone in UTIs, with ampicillin having the highest resistance of 82.5% [27]. Shirvani et al. revealed that 48.5% of the isolates were resistant to ceftriaxone, and the highest rate was for nalidixic acid at 73.5%) [28]. Further evidence of ceftriaxone resistance in Jordan is provided by surveillance data from the Islamic Specialized Hospital (ISH), which reported a consistent 40% resistance rate of *E. coli* between 2020 and 2022. This aligns with the current study’s findings and highlights a concerning trend in resistance to third-generation cephalosporins used empirically [29].

In this study, the female sex was a predictor of isolates with higher resistance. This is contrary to the results of Gu et al.’s study in patients with urinary stones, where *E. coli* isolates from females showed a higher susceptibility to most antibiotics than those from males [30]. Linhares et al. examined resistance patterns in community-acquired UTIs over a ten-year period and found that bacterial isolates from female patients exhibited lower resistance than those from male patients [31]. Alrabayah et al. conducted a 10-year retrospective study to assess the antimicrobial resistance trends of *E. coli* isolates in urine cultures of women; the resistance to ceftriaxone was 65.7%, which is higher than that identified in our study. This might coincide with our finding that the resistance of *E. coli* is higher in women than in men, and that the rate would be higher in a study that evaluated only women [32]. Additionally, females are more likely to experience UTIs than males, and their increased exposure to antibiotics could contribute to the higher prevalence of resistance observed in this group [33].

Previous results suggest that the choice of antimicrobial therapy should consider the sex of the patient, which might be challenging when susceptibility in sexes differs depending on the type of antibiotic clinical setting (outpatient/hospitalized, ICU/non-ICU) [31,34].

This study sheds light on the resistance patterns of an important antimicrobial-resistant pathogen in Jordan, *E. coli*, and is the first to examine its susceptibility patterns in UTIs in both men and women. It was conducted in a large tertiary education hospital that accepts referrals from other hospitals.

However, several limitations should be considered. First, as a retrospective analysis based on electronic medical records, some data were missing, and certain variables might have led to the outcomes not being consistently documented. Notably, data on the timing of infection onset were not available, limiting our ability to distinguish between hospital-acquired and pre-existing infections. Second, the study was conducted within a single institution, meaning the findings may not fully reflect practices or outcomes at other hospitals, potentially limiting their generalizability to different settings or regions. Third, data were collected specifically from patients in the internal medicine department, which may not represent the broader hospital population, further limiting the applicability of the results.

## 5. Conclusions

Antibiotic resistance surveillance is essential for combating antimicrobial resistance. UTI isolates of *E. coli* had the highest susceptibility to imipenem/cilastatin and the highest resistance to ceftriaxone. A higher rate of resistant isolates was observed in females than in males, suggesting that antibiotic treatment should be tailored based on patient traits such as sex, age, comorbidities, and the previous use of antibiotics. Wise and appropriate use of empirical therapy is recommended and can be achieved through regular assessment of common pathogens and their susceptibility trends in medical institutions. Future studies should focus on improving the accuracy of empirical antibiotic prescriptions, optimizing sensitivity testing protocols, and addressing gender-based disparities in resistance patterns to reduce treatment failure and complications of antimicrobial resistance.

## Figures and Tables

**Table 1:** Demographic and medical characteristics of the study sample (n= 230).

Parameter	Median (IQR)	n (%)
Age (years)	64.0 (25.0)	
Age categories (years)		
◦ 20–24.9		4 (1.7)
◦ 25–34.9		22 (9.6)
◦ 35–44.9		16 (7)
◦ 45–54.9		31 (13.5)
◦ 55–64.9		44 (19.1)
◦ ≥65		113 (49.1)
Gender		
◦ Female		166 (72.2)
◦ Male		64 (27.8)
Number of chronic medications		
◦ 0–1		50 (21.7)
◦ 2–3		58 (25.2)
◦ ≥4		122 (53)
Length of Stay	10.0 (9.0)	

IQR: interquartile range.

**Table 2:** Microorganism distribution and antibiotic prescriptions in patients with UTIs (n = 230).

Parameter	Mean ± SD	n (%)
Number of microorganisms per culture		
◦ One		187 (81.3)
◦ Two		40 (17.4)
◦ Three		3 (1.3)
Total number of identified micro-organisms		276
Mean number of identified microorganisms per patient	1.2 ± 0.4	
Number of prescribed antibiotics per patient		
◦ Zero		29 (12.6)
◦ One		170 (73.9)
◦ Two		27 (11.7)
◦ Three		4 (1.7)
Total number of prescribed empiric antibiotics		236
Number of empiric antibiotics prescribed per patient	1 ± 0.5	

SD: standard deviation.

**Table 3:** Susceptibility testing results for antibiotics prescribed to patients with *E. coli* urinary tract infections (n = 230).

Parameter	n (%)
Patients without empiric treatment	29 (12.6)
Patients prescribed antibiotics not covering *E. coli*	8 (3.5)
Availability of susceptibility reporting among patients receiving antibiotics covering *E. coli*	
◦ Report available	136 (59.1)
◦ Report not available	59 (24.8)
Susceptibility of *E. coli* among patients	
◦ Resistant *E. coli*	62 (27.0)
◦ Sensitive *E. coli*	74 (32.1)

**Table 4:** Summary of antibiotic sensitivity for and resistance to *E. coli* among prescribed empiric treatments (n = 236).

Parameter	n (%)
Antibiotics without sensitivity reports	72/236 (30.5)
Antibiotics not covering *E. coli*	8/236 (3.4)
Antibiotics to which *E. coli* is sensitive ◦ Imipenem/Cilastatin ◦ Levofloxacin ◦ Piperacillin/Tazobactam ◦ Cefuroxime ◦ Others	89/236 (37.7) 43/89 11/89 9/89 7/89 19/89
Antibiotics to which *E. coli* is sensitive ◦ Ceftriaxone ◦ Levofloxacin ◦ Cefuroxime ◦ Imipenem/Cilastatin ◦ Others	67/236 (28.4) 32/67 15/67 10/67 2/67 8/67

**Table 5:** The impact of antibiotic sensitivity testing on the length of hospital stay (n = 195).

Outcome	Sensitivity Test Present (n = 136)	Sensitivity Test Absent (n = 57)	*p*-Value #
Length of stay (days), median (IQR)	10.0 (10.0)	8.0 (6.0)	0.032

# Using the Mann–Whitney U test.

**Table 6:** Association of gender and length of hospital stay with antibiotic resistance among *E. coli* isolate (n = 136).

Variables	Resistant Isolates	Sensitive Isolates	*p*-Value Statistical Test
Gender			
Male (n = 39)	12 (30.8%)	27 (69.2%)	0.036
Female (n = 97)	50 (51.5%)	47 (48.5%)	Pearson Chi-Square
Length of stay (median, IQR)	11 (10)	9.5 (8)	0.190 Mann–Whitney U Test

Significant at the level of 0.05. IQR: interquartile range.

## Data Availability

The data that support the findings of this study are available from [Rana Abu-Farha] upon reasonable request due to privacy/ethical restrictions.

## Abbreviations

The following abbreviations are used in this manuscript:

UTI	Urinary Tract Infection
*Escherichia coli*	*E. coli*
ESBLs	Extended-Spectrum Beta-Lactamases
MDR	Multidrug-Resistant
JUH	Jordan University Hospital
WHO	World Health Organization
ICU	Intensive Care Unit
